# Editorial for Special Issue: “Current and Emerging Therapies for Lower Urinary Tract Symptoms and Benign Prostatic Hyperplasia”

**DOI:** 10.3390/jcm13092453

**Published:** 2024-04-23

**Authors:** Eliophotos Savvides, Georgios Langas, Petros Sountoulides

**Affiliations:** 1 Department of Urology, Main Kinzig Kliniken, 63571 Gelnhausen, Germany; 2 1st Department of Urology, Faculty of Health Sciences, Medical School, Aristotle University of Thessaloniki, 54124 Thessaloniki, Greece; glag93@hotmail.com

Lower urinary tract symptoms (LUTS) caused by benign prostatic hyperplasia (BPH) constitute a significant health concern worldwide, particularly among aging male populations. 

Over the years, therapeutic options for managing LUTS caused by BPH have significantly evolved, with the aim of alleviating symptoms, improving urinary flow, and preventing disease progression. Current management options begin with conservative approaches to lifestyle modifications with or without pharmacotherapy and can escalate to minimally invasive surgical treatments or even open prostatectomy. However, emerging research continues to explore novel therapeutic modalities to enhance treatment efficacy, minimize adverse effects, and offer customized care to patients. By synthesizing existing knowledge and recent advancements, this Special Issue aims to inform clinicians and researchers of the evolving landscape of therapeutic options for LUTS and BPH. Understanding the mechanisms of action and the comparative effectiveness of current and emerging therapies is crucial for optimizing patient outcomes, enhancing quality of life, and addressing the growing burden of LUTSs and BPH on healthcare systems worldwide.

Naiyila et al. [1] contributed an excellent review to this Special Issue entitled “A Novel Insight into the Immune-Related Interaction of Inflammatory Cytokines in Benign Prostatic Hyperplasia”, elucidating the connection between chronic inflammation induced by various stressors—such as bacterial and viral infections, dietary factors, hormones, and urinary reflux—and benign prostatic hyperplasia (BPH). Specifically, this review delves into the pathophysiological mechanisms and interactions involving various cytokines, including IL-8, IL-15, and TGF-β, and their impact on prostate gland size. Growth factors released by stromal cells in the prostate intricately regulate prostate cell dynamics through autocrine and paracrine signaling. In benign prostatic hyperplasia (BPH), fibroblasts expressing an androgen receptor (AR) exhibit an overexpression of fibroblast growth factor-2 (FGF-2) and fibroblast growth factor-7 (FGF-7), contributing to disease pathogenesis. Transforming growth factor-β1 (TGF-β1) induces the differentiation of mesenchymal fibroblasts into myofibroblasts, thereby promoting hyperplasia associated with BPH. Elevated levels of growth factors correlate with BPH occurrence. Recent research highlights the role of pro-inflammatory macrophages in inducing interstitial hyperplasia in BPH through the AR signaling pathway. Studies employing co-culture systems of human macrophages and prostate mesenchymal cells demonstrate increased macrophage infiltration and AR expression in the transitional zone compared to the peripheral zone. Furthermore, inflammatory signaling pathways, particularly the stromal AR/CCL3/stromal cell expansion pathway, synergize with AR signaling to promote prostate stromal cell proliferation. Moreover, chronic or acute inflammation is believed to induce proliferative events and post-translational DNA alterations in prostate tissue, primarily through oxidative stress (OS) pathways. OS arises from an imbalance between reactive oxygen species (ROS) production and the cellular antioxidant defense system, leading to cellular damage and dysfunction. High ROS levels cause significant damage to proteins, fats, and DNA, exacerbating oxidative damage when antioxidant defenses are compromised. OS can directly stimulate cell proliferation via MAP kinase and PI3K/AKT signaling pathways, while DNA oxidative damage can induce cell senescence and cytokine production. Some studies demonstrate elevated levels of oxidative stress markers in BPH tissue, correlating with prostate enlargement. Moreover, animal models show increased lipid peroxidation and decreased antioxidant enzyme activity in BPH. Monitoring oxidation levels and antioxidant concentrations can aid BPH diagnosis, with antioxidant therapy showing promise in enhancing treatment efficacy. Overall, inflammation-induced oxidative stress plays a crucial role in BPH pathogenesis, highlighting its significance in prostate cell growth and tissue destruction. Normal prostate tissue harbors a significant population of immune cells, predominantly T lymphocytes, with CD8+ T cells primarily located in the periglandular regions and CD4+ T cells distributed in the stroma. Clinical evidence, including findings from the Medical Therapy of Prostatic Symptoms (MTOPS) study, highlights the association between chronic inflammation and BPH/LUTS severity. Chronic inflammation, often triggered by various stimuli, including infections, dietary factors, hormones, and urine reflux, leads to tissue damage and the release of pro-inflammatory cytokines and growth factors. This inflammatory microenvironment contributes to the excessive proliferation of BPH stroma, akin to the wound healing processes observed in fibrosis-related diseases. Understanding inflammation-induced tissue injury as a chronic wound healing process sheds light on potential therapeutic targets for managing BPH. These findings underscore the significance of inflammation in BPH progression, suggesting potential targets for therapeutic intervention. Potential causative therapies that are currently available or in development are briefly analyzed, elucidating their respective mechanisms of action. The significance of phytotherapy and PDE5-I in particular is explored, since these interventions regulate and mitigate local inflammation, effectively controlling prostate growth.

Furthermore, within the broader spectrum of therapeutic interventions for benign prostatic hyperplasia (BPH), in an exhaustive review, Antoniou et al. [2] rigorously examine the utilization of phytotherapeutic agents, including serenoa repens, cucurbita pepo, pygeum Africanum, etc. Employing a holistic approach, this review delves into the mechanisms of action and origin these agents, as well as providing a meticulous analysis of their efficacy and safety profiles. The exceptional importance of this review is underscored by prevailing trends, where nearly two-thirds of individuals aged over 70 incorporate dietary supplements into their regimen. Antoniou et al. evaluate current evidence and presents a pragmatic approach that provides insights into the various forms in which these substances are readily available. Cumulatively, the role of phytotherapy is appraised in accordance with the guidelines set forth by the European Association of Urology (EAU) and the American Urological Association (AAU). The most researched substance on that list is serenoa repens, which is somewhat recommended by EAU Guidelines as a treatment for patients with mild symptoms. The current limitations of their application are acknowledged, with the authors recognizing that the principal merit of their research lies in the minimal adverse events experienced by patients, despite the ongoing debate surrounding their efficacy.

Analyzing the role of phytotherapy in the treatment of benign prostatic hyperplasia (BPH), with a particular focus on serenoa repens monotherapy and its combination with other conventional BPH medications, De Nunzio et al. [3] conducted an extensive review that provides practical solutions. Their review also suggests which subset of patients can benefit the most from combination therapy. The rationale behind employing combination therapy, already widely adopted by urologists in various countries, stems from the observation that monotherapy with standard doses, while effective, comes with significant side effects. These include sexual disturbances, gynecomastia, hypotension, and reduced libido. By incorporating combination therapies and subsequently reducing the doses of the active drugs, De Nunzio et al. suggest that the aforementioned adverse events can be minimized while maintaining similar levels of efficacy. The authors underscore the role of local inflammation in BPH and propose that serenoa repens, known for its anti-inflammatory properties—as demonstrated in a randomized control trial using a prostate biopsy in patients pre- and post-serenoa repens—leads to a lower inflammation grade as demonstrated by the reduced number of infiltrated inflammatory cells. In summary, combining the hexanic extract of serenoa repens (HESr) with α-blockers demonstrates comparable efficacy with α-blockers alone or in conjunction with 5-ARIs for treating LUTS/BPH, with significantly fewer sexual side effects. Given patients’ preference for lower-risk options with fewer sexual adverse effects, this type of combination therapy, targeting storage symptoms such as urgency and incontinence, is clinically relevant. Real-world evidence supports HESr + α-blocker combination therapy as a frequently adopted strategy of first-line treatment, particularly in older patients with established LUTS/BPH. Patient selection for combination therapy, especially those with a specific clinical profile such as prostatic inflammatory status (PIS), is crucial for optimizing treatment responses. However, current research lacks robust randomized clinical trials on HESr combination therapies, highlighting the need for more comprehensive studies to guide personalized treatment decisions. The synthesis of existing data underscores the necessity for further research to refine HESr + α-blocker combination treatments, ultimately enhancing patient outcomes and quality of life. Apart from this category, patients with comorbidities such as diabetes mellitus, higher BMI, and larger prostate volume may also benefit. As physicians, it is our duty to assist patients in receiving the best medication therapy based on their preferences, and in some cases, a combination therapy containing phytotherapy elements is advisable.

LUTS caused by benign prostatic obstruction (BPO) and bladder tumors can often coexist, particularly among elderly patients. However, concerns regarding tumor cell seeding and recurrence in the prostatic urethra have led many surgeons to avoid performing the transurethral resection of bladder tumors (TURBT) and endoscopic surgery for BPO concurrently. Through a systematic review and meta-analysis, Savvides et al. [4] aimed to evaluate oncological safety and impact on patients’ quality of life associated with concomitant TURBT and endoscopic BPO surgery. The researchers conducted a thorough search of various databases and sources for relevant studies. They included three randomized trials and twelve retrospective observational studies involving 2421 participants. The meta-analysis, utilizing random-effects models, compared concomitant TURBT and BPO surgery with TURBT alone in terms of bladder tumor recurrence and progression rates. The findings from studies with good methodological quality revealed no statistically significant differences in overall bladder tumor recurrence rates between concomitant surgery and TURBT alone. Similarly, there were no significant differences observed in recurrence rates specifically located at the bladder neck and/or prostatic urethra, time to first recurrence, or progression rate. Subgroup analyses based on tumor grade, number of tumors, and post-TURBT single-instillation chemotherapy did not yield significant differences in overall bladder tumor recurrence. However, the level of evidence for all outcomes was estimated to be low. Despite the lack of significant differences in bladder tumor outcomes, concomitant surgery was associated with an improvement in LUTS-related quality of life. Thus, the study concludes that concomitant endoscopic BPO surgery and TURBT are oncologically safe procedures that can enhance the quality of life of patients experiencing LUTS associated with BPO and bladder tumors.

In conclusion, the pathophysiological mechanisms underlying BPH and inflammation are exceedingly intricate, involving a multitude of cellular and molecular interactions within the prostate gland. Both BPH and prostatic inflammation significantly contribute to LUTS and can exacerbate each other’s effects, further complicating the clinical management of patients. In addressing these complexities, phytotherapy emerges as a promising adjunct to the treatment of BPH and prostatic inflammation. Phytotherapeutic agents, such as serenoa repens, offer a diverse array of bioactive compounds with potential therapeutic benefits, including anti-inflammatory and anti-proliferative properties. Whether utilized as monotherapy or in combination with conventional pharmacotherapy, phytotherapy presents a valuable therapeutic avenue for mitigating symptoms and improving patient outcomes in BPH. Moreover, there is sufficient evidence regarding the safety of performing concomitant TURBT and TURP. Despite concerns regarding tumor cell seeding and recurrence, evidence suggests that simultaneous surgical interventions for BPO and bladder tumors do not significantly increase the risk of oncological adverse events. Moving forward, continued research efforts are warranted to deepen our understanding of the intricate pathophysiology of BPH and inflammation, elucidate the mechanisms of action of phytotherapeutic agents, and refine surgical techniques to optimize patient outcomes. By integrating these insights into clinical practice, healthcare providers can offer more comprehensive and personalized management strategies for patients with BPH and the associated prostatic inflammation, ultimately enhancing their quality of life and clinical outcomes.
